# Study on Early Hydration Mechanism of Double-Liquid Grouting Material Modified by Composite Early Strength Agent

**DOI:** 10.3390/ma16196475

**Published:** 2023-09-29

**Authors:** Xinming Chen, Jie Wang, Huazhe Jiao, Zhi Yang, Diantao Zheng, Jinyu Sun

**Affiliations:** 1 Henan Key Laboratory of Underground Engineering and Disaster Prevention and Control, Henan Polytechnic University, Jiaozuo 454150, China; chenxinming@163.com (X.C.); jiaohuazhe@hpu.edu.cn (H.J.); 212108020060@home.hpu.edu.cn (Z.Y.); 212208020076@home.hpu.edu.cn (D.Z.); 15690765597@163.com (J.S.); 2 College of Civil Engineering, Henan Polytechnic University, Jiaozuo 454150, China

**Keywords:** grouting material, early strength agent, hydration mechanism, calcium formate, sodium sulfate, lithium carbonate

## Abstract

To achieve an adjustable setting time and significantly improved early strength of a new type of sulphoaluminate cement-based double-liquid grouting material (SACDL), the effects of calcium formate, sodium sulfate, lithium carbonate, and a composite early strength agent on the setting hardening and early hydration behavior of SACDL paste were studied by means of setting time, fluidity, compressive strength, and viscosity tests. The results showed that the adsorption and osmosis of calcium formate, the complex decomposition of sodium sulfate, the precipitation polarization of lithium carbonate and the synergistic action of the composite early strength agent could accelerate the early hydration rate of SACDL, shorten the coagulation time, and improve the early strength of SACDL. The composite effect of 0.8% calcium formate and 0.5% sodium sulfate is the most significant in promoting coagulation and early strength; the initial setting time and final setting time of the slurry were shortened to 5 min and 10 min, respectively; and the 3 h compressive strength was capable of reaching 16.7 MPa, 31% higher than that of the blank group. In addition, X-ray diffraction and SEM morphology observation were used to study the composition of the hydration products and the evolution of the microstructure, which revealed the early hydration mechanism of SACDL under the synergistic effect of the composite early strength agent: (1) The solubility of tricalcium aluminate (C_3_A) and dihydrate gypsum (CaSO_4_·2H_2_O) increased under the low content composite early strength agent condition, which increased the ettringite (AFt) formation rate. HCOO^−^ was able to penetrate the hydration layers of tricalcium silicate (C_3_S) and dicalcium silicate (C_2_S), accelerating the dissolution of C_3_S and C_2_S and promoting the early hydration of SACDL. (2) Under the condition of a high dosage of the composite early strength agent, the further increase in Ca^2+^ concentration promoted the crystallization nodules and precipitation of CH and accelerated the formation of calcium silicate hydrate (C-S-H) gel. C-S-H was filled between a large number of rod-like AFt crystals, thus making the structure more dense.

## 1. Introduction

Grouting material is an important factor in ensuring the quality of grouting, and in recent years, with the continuous expansion of the scale of engineering construction, underground space development, and the complexity and diversity of engineering geological conditions, grouting material requirements are more stringent, which promotes the continuous development of grouting materials [1,2]. SACDL is a new type of sulfoaluminate cement-based two-fluid grouting material, which is mainly divided into the A material and B material, where the A material comprises a sulfoaluminate cement clinker and the B material consists of gypsum, quicklime, slaked lime, etc. A and B materials contain a certain amount of ethylene vinyl acetate copolymer (VAE) emulsions, polycarboxylic acid water reducer, hydroxypropyl methylcellulose (HPMC), and many other additives. With SACDL, A liquid and B liquid can be placed separately to ensure that they do not solidify for a long time, while the A and B liquids can react quickly after mixing. The grouted stone body has high strength and good durability; thus, it has a wide range of application prospects in mining, dams, tunnels, slopes, bridges, foundation pits, and many other engineering fields [3,4]. In an emergency repair project, the grouting material condensation speed was fast and the early strength was high, controlling the deformation of the surrounding rock quickly. Therefore, it is especially important to accelerate the grouting material’s condensation time and improve its early strength [5].

In order to meet the needs of the practical engineering field, researchers are constantly looking for new early strength agent materials to improve the early performance of grouting materials. Some studies have focused on the use of graphene oxide [6], nano-TiO_2_ [7,8], nano-CaCO_3_ [9,10], composite early strength agents [11,12], and other new materials to be used as early strength agents. These materials have high surface activity and catalytic effects, which can accelerate the hydration reaction of cement and improve the early strength. Among them, nano-calcium carbonate (NC) has a significant effect on the early strength of cement. NC can promote the formation of calcium–silicate–hydrate (C-S-H) gel and hydrated calcium carbon aluminate through nucleation and chemical reactions, thereby accelerating cement hydration [13,14,15]. Some researchers have added composite early strength agents to cement-based materials and achieved some remarkable results. Haijun Zhou et al. [16] investigated the effects of calcium formate, aluminum sulfate, lithium carbonate, and triethanolamine on the performance of sulfoaluminate cement-based ultra-high-performance concrete (UHPC), and the results showed that the four types of early strength agents shortened the setting time of UHPC and significantly increased its early strength; the 3d compressive strength reached 88.7 MPa. Yu Xin et al. [17] used in situ XRD and isothermal calorimetry to study the effect of the inorganic salt early strength agents sodium thiocyanate, sodium sulfate, and sodium thiosulfate on the early hydration of cement, and found that the addition of three kinds of inorganic salt early strength agent within the appropriate dosing range can promote the hydration reaction of the clinker minerals C_3_S, C_3_A, and C_4_AF to varying degrees to accelerate the rate of the exothermic rate of hydration of the cement and, thus, increase the degree of early hydration of the cement. Jian Wang and others [12] believe that lithium has a greater effect on the early hydration of cement, and 0.3‰ lithium can effectively promote the early hydration of cement and improve the early strength of cement. Lin Min et al. [18] developed a new inorganic class of composite early strength agent, triethanolamine. Sodium sulfate, with a certain proportion of the composite blended into cement stabilized crushed stone, can effectively achieve early strength improvement and improve the anti-shrinkage performance of cement-stabilized crushed stone.

It can be seen that organic salt calcium formate, inorganic salt sodium sulfate, and lithium carbonate can all have effects on the properties and hydration patterns of cement. Based on this, in order to realize the adjustable condensation time of SACDL and a significant increase in early strength, this paper selects calcium formate and sodium sulfate to configure an organic–inorganic composite early strength agent and investigates the influence law of calcium formate, sodium sulfate, lithium carbonate, and composite early strength agents on the early hydration of SACDL.

## 2. Experimental Section

### 2.1. Raw Materials

(1) A material: the choice of sulfoaluminate cement clinker, from Zhengzhou City Jianwen Special Material Technology Co., Ltd. (Zhengzhou, China) production, with a setting time of 5–6 min, 1 d strength of 35 MPa, and 3 d strength of 45 MPa. The specific chemical composition is shown in Table 1.
(2) B material: gypsum hemihydrate (CaSO_4_·0.5H_2_O) from Hongli Gypsum Factory in Quwo County, China. Chemical composition is shown in Table 1. Quicklime was obtained from the Yiyang Taojiang Lime Factory (Yiyang, China), and the fineness (10 mesh throughput) was 95.89%. The product was tested and met the requirements of national industry standards, such as GB/T8576-2010. Slaked lime was obtained from Ganzhou Fangsheng Building Materials Lime Factory Limited (Ganzhou, China), and the fineness (200 mesh throughput) was 96.14%.

(3) Admixtures: VAE emulsion, polycarboxylic acid water reducing agent (PBS), HPMC, borax (BX), sodium citrate (SCE), calcium formate, sodium sulfate, and lithium carbonate were analytically pure reagents. Among them, calcium formate was derived from Tianjin Damao Chemical Reagent Factory (Tianjin, China) as a white crystalline powder with a molecular weight of 130.11 and a content ≥ 98%. Sodium sulfate was obtained from Tianjin Dengfeng Chemical Reagent Factory (Tianjin, China) as a white crystalline powder with a molecular weight of 142.04 and a content ≥ 99%. Lithium carbonate was obtained from Xilong Science Co., Ltd. (Shantou, China) as a lightweight white powder, with a molecular weight of 73.89 and a content ≥ 98%.

### 2.2. Pilot Program

Liquid A and liquid B were formulated as shown in Table 2, keeping the proportion of each component unchanged, mixing the early strength agent in liquid B, and mixing liquid A and liquid B at a ratio of 1:1 to configure SACDL slurry. The test was divided into blank, single-doped, and composite groups, in which the blank group was not doped with an early strength agent and the single-doped group was doped with calcium formate (0.8%, 1.2%, and 1.6%), sodium sulfate (0.8%, 1%, and 1.2%), and lithium carbonate (0.12%, 0.15%, and 0.18%), respectively, individually in the B liquid. In the composite group, calcium formate and sodium sulfate were added in certain proportions, and the compound dosage of calcium formate and sodium sulfate was determined based on a two-factor, three-level experiment, as shown in Table 3.

### 2.3. Test Methods

(1) Determination of setting time: The Vicat meter produced by Zhejiang Dadi Instrument Co., Ltd. (Wenling, China) was used to determine the setting time of the slurry, and the specific determination method refers to the provisions of GB/T 1346-2011.
(2) Flowability test: A 60 × 36 × 60 mm net slurry mold produced by Cangzhou Zhulong Engineering Instrument Co., Ltd. (Cangzhou, China) was used to determine the flowability of the B liquid, and the specific determination method referred to the provisions of GB/T 8077-2012.
(3) Compression test: The compressive strength of the mortar specimens at specific ages (3 h, 6 h, 1 d, 3 d, 7 d, and 28 d) was tested using a hydraulic press manufactured by Wuxi Building Material Testing Instrument Co., Ltd. (Wuxi, China) The specific determination method was operated in accordance with the provisions of GB/T 17671-2021.
(4) Viscosity test: The U.S. Bohlefeld Brookfield DV2TLV viscometer (BROOKFIELD, Massachusetts, USA.)was used, and the No. 6 rotor and RTD temperature probe were selected for real-time monitoring of the viscosity of the ethyl-liquid and temperature. The rotor speed was set to 6.0 RPM.
(5) Scanning electron microscope (SEM) test: The British Cressington automatic ion sputtering instrument 108AUTO was used on the surface of the sample to form a conductive layer of gold spraying, improving the conductivity and stability of the sample. A German Merlin Compact (Carl Zeiss AG, Oberkochen, Germany) scanning electron microscope was selected under low vacuum conditions, and the microstructure of the sample hydrated for 3 h was observed using secondary electron (SE) mode at an accelerating voltage of 15.0 kV.
(6) X-ray (XRD) diffraction test: Quantitative analysis of the hydration of the 3 h sample was performed using the SmartLab rotating-target X-ray diffractometer (Japan Neo- Co., Ltd. Tokyo, Japan). The scanning range was set from 5° to 65°, with a scanning rate of 0.04°/s.

## 3. Results and Discussion

### 3.1. Effect of Early Strength Agent on Performance of SACDL

#### 3.1.1. Effect of Early Strengthening Agents on SACDL Setting Time

**Figure 1:**
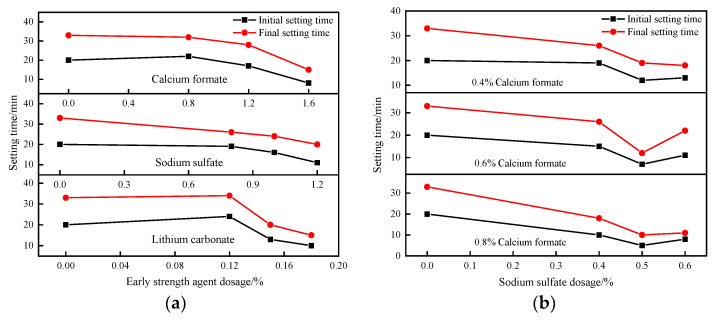
The effect of single and composite early strength agents on the setting time of SACDL. (**a**) Setting time of SACDL single-doped early strength agent; (**b**) setting time of composite early strength agent SACDL.

The effects of the three early strength agents—calcium formate, sodium sulfate, and lithium carbonate—doped alone on the setting time of SACDL, are shown in Figure 1a. The SACDL slurry setting time was shortened with the increase in dosages of three kinds of early strength agents, and the coagulation-promoting effect was in the order of calcium formate > sodium sulfate > lithium carbonate. The optimal dosages were 1.6% (Ca(HCOO)_2_), 1.2% (Na_2_SO_4_), and 0.18% (Li_2_CO_3_), respectively. As shown in Figure 1a, when the dosages of calcium formate and lithium carbonate were low, the coagulation-promoting effect was not obvious, and the coagulation time of the slurry decreased sharply with the increase in the dosage. When the dosage of calcium formate was increased to 1.6%, the durations of the initial coagulation and the final coagulation of the slurry were shortened to 8 min and 15 min from 20 min and 33 min in the blank group, respectively, and the effect of promoting coagulation was significant. For sodium sulfate, the slurry setting time was shortened with the increase in the sodium sulfate dosage. The trend of the curve was relatively smooth because, with the increase in the sodium sulfate dosage, the concentration of NaOH in the hydration reaction increased accordingly. At this time, NaOH as an active agent further improved the solubility of C_3_A and CaSO_4_·2H_2_O to increase the rate of generation of AFt, leading to the rapid agglomeration and hardening of the SACDL [19,20].

From Figure 1b, it can be seen that when the dosages of calcium formate were 0.4%, 0.6%, and 0.8%, compounding sodium sulfate was able to effectively shorten the setting time of SACDL slurry. The setting time of the slurry shortened with the increase in the dosage of calcium formate, and the effect of promoting the setting of the slurry was most obvious when the dosage of calcium formate was 0.8%. When the dosage of calcium formate was certain, the slurry setting time was shortened and then prolonged with the increase in sodium sulfate dosage. The 0.5% sodium sulfate was the best composite content, and the initial setting time and final setting time of the slurry were shortened to 5 min and 10 min, respectively. When the content of sodium sulfate increased to 0.6%, the setting time of the slurry was significantly prolonged, which is due to the fact that sodium sulfate easily reacts with Ca^2+^ dissociated from calcium formate. With the increase in the dosage of sodium sulfate, the content of CaSO_4_·2H_2_O in the slurry increased significantly, leading to a substantial increase in the formation of ettringite. The needle-like calcium ettringite interlocked between cement particles, forming a protective layer that hindered the hydration rate [19,21,22].

#### 3.1.2. Effect of Early Strength Agent on the Flowability of B-Liquid

**Figure 2:**
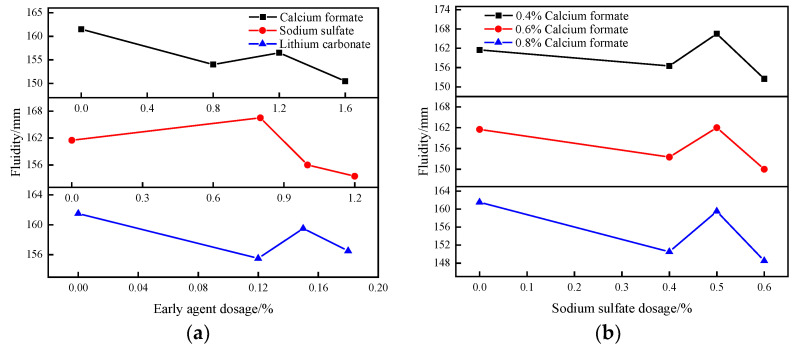
The effect of single-doped and composite early strength agents on the fluidity of SACDL. (**a**) SACDL fluidity with single-doped early strength agent; (**b**) SACDL fluidity with composite early strength agent.

Figure 2a shows the effect of single doping of early strength agents on the flowability of the B liquid. Calcium formate and lithium carbonate had inhibitory effects on the fluidity of liquid B in the dosage range, and the slurry’s flowability with the increase in doping showed a decreasing trend, followed by increasing and then decreasing. The low content of calcium formate and lithium carbonate was able to increase the amount of precipitates in the reaction system and reduce the fluidity of the slurry [23,24]. With the increase in the contents of the two, the viscosity and surface tension of the cement paste gradually decreased, thereby reducing the adsorption and friction between the cement particles and improving the fluidity of the slurry. The high content of calcium formate and lithium carbonate greatly increased the formation rate of precipitates such as AFt and CaCO_3_ in the reaction system, hindered the flow and dispersion of the cement paste, and led to an additional decrease in fluidity [25,26,27]. The fluidity of the slurry reached the minimum under the action of 1.6% calcium formate, which decreased from 162 mm in the blank group to 151 mm. The optimum dosage of sodium sulfate was 0.8%, and the fluidity extended to 167 mm. When the dosage of sodium sulfate was greater than 0.8%, the fluidity of the liquid decreased with the increase in the dosage of sodium sulfate. When the dosage was 1.2%, the fluidity decreased to 154 mm. It can be seen from Figure 2b that when the amount of calcium formate was constant, the fluidity of the liquid also first showed a trend of decreasing, then increasing and decreasing again with the increase in the amount of sodium sulfate. When the content of calcium formate was 0.8% and the content of sodium sulfate was 0.6%, the fluidity of the liquid decreased to 149 mm. Under the action of a high level of the composite early strength agent, the content of CaSO_4_ in the reaction system increased significantly, and the crystallization reaction of CaSO_4_ in cement and the solid products in the crystallization process easily occupied the pores of cement particles, resulting in a significant decrease in the slurry’s fluidity [28,29].

#### 3.1.3. Effect of Early Strength Agent on SACDL Strength

**Figure 3:**
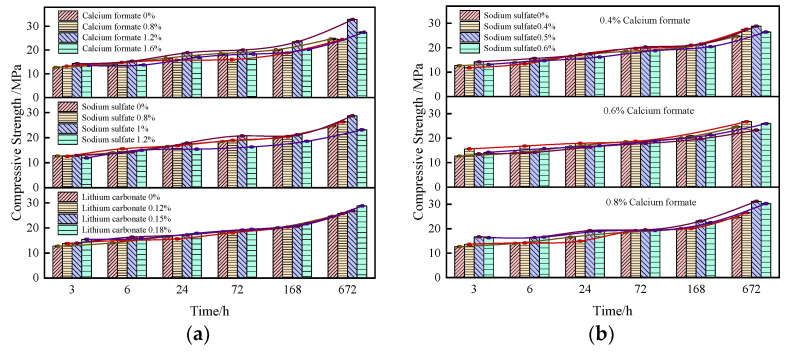
Effect of single and composite early strength agents on the strength of SACDL. (**a**) SACDL strength with single-doped early strength agent; (**b**) SACDL strength with composite early strength agent.

The compressive strength of SACDL under the action of single-mixed early strength agents at the ages of 3 h, 6 h, 1 d, 3 d, 7 d, and 28 d are shown in Figure 3a. The test results show that the compressive strength of SACDL increased and then decreased according to the dosage of calcium formate, which was optimal at 1.2%. At this dose, the early strength of SACDL was improved under the condition of ensuring the substantial increase in its late strength. The 3 h and 28 d compressive strengths reached 14.3 MPa and 32.8 MPa, 12.3% and 33.2% higher than those of the blank group, respectively. Sodium sulfate’s enhancement effect on the 3 h compressive strength of SACDL was not obvious. At 6 h and after the strength improved compared to that of the blank group, 1% was achieved for the best dosage of sodium sulfate, and the 3 d compressive strength reached up to 20.8 MPa. As shown by the growth trend curve, SACDL’s compressive strength increased with the increase in lithium carbonate doping. The strength enhancement effect was best when the lithium carbonate doping was increased to 0.18%, and the 3 h strength reached 15.4 MPa.

With contents of calcium formate of 0.4%, 0.6%, and 0.8%, the effect of composite sodium sulfate on the compressive strength of SACDL is shown in Figure 3b. When the dosage of calcium formate was 0.4%, the compounding of 0.5% sodium sulfate was the most favorable for the late strength of the calculi, and when the dosage of calcium formate was increased to 0.6%, the compounding of 0.4% sodium sulfate had a significant effect on the enhancement of the early strength of the calculi. When the dosage of calcium formate reached 0.8%, at this time, compounding with sodium sulfate had the best effect on the strength enhancement of SACDL; the strength of the nodule body did not change much compared with that of the blank group when compounding with 0.4% sodium sulfate. With the increase in sodium sulfate doping, the compressive strength increased significantly, and the best effect was achieved when the sodium sulfate doping was 0.5%. The 3 h and 28 d compressive strengths reached 16.7 MPa and 31.2 MPa, respectively, 31% and 26.4% higher than those of the blank group, and the strength enhancement effect was significantly higher than that of the single-doped group.

#### 3.1.4. Time-Varying Characteristic Curve of Viscosity and Temperature of B Liquid under the Action of Early Strength Agents

**Figure 4:**
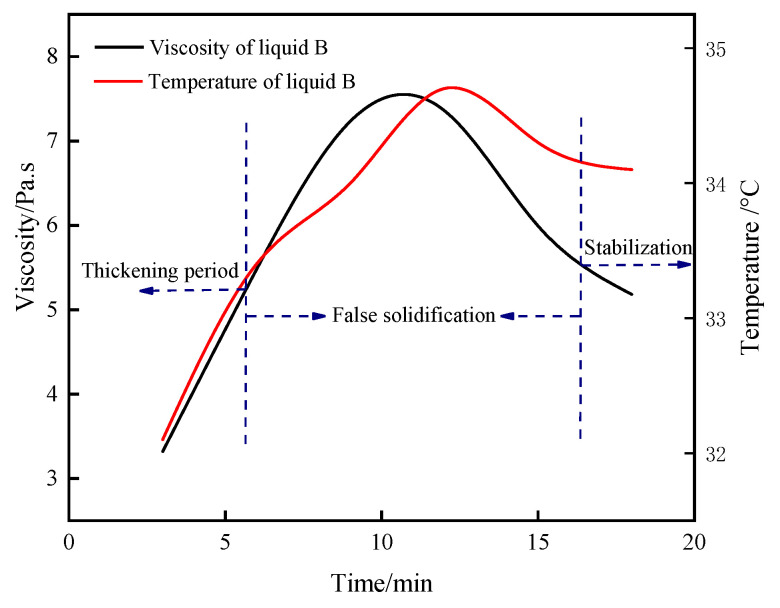
Time-varying characteristic curve of B liquid’s viscosity and temperature.

Viscosity reflects the magnitude of viscous resistance to fluid flow; the lower the viscosity of the slurry, the better its injectability [30,31]. The curves of the viscosity and temperature of the B liquid with the hydration time are shown in Figure 4. The hydration of CaSO_4_·0.5H_2_O is an exothermic process, and the viscosity and temperature can both respond to the degree of CaSO_4_·0.5H_2_O hydration while the time-varying characteristic curves of the two basically remain the same [32,33,34]. According to the viscosity, the changes can be divided into “thickening period”, “false solidification”, and “stabilization” of the three stages of the hydration reaction of the B liquid. In the thickening phase, as the hydration reaction proceeded, the viscosity of the B-liquid continued to grow until the slurry’s mobility was substantially lost and it began to show the characteristics of coagulation. The viscosity of the slurry at this stage basically increased in a positive proportion, which was due to the reaction of CaSO_4_·0.5H_2_O dissolved in water to form CaSO_4_·2H_2_O, as shown in Equation (1):

(1) $$CaSO_{4}\cdot 0.5H_{2}O+1.5H_{2}O\to CaSO_{4}\cdot 2H_{2}O$$

**Figure 5:**
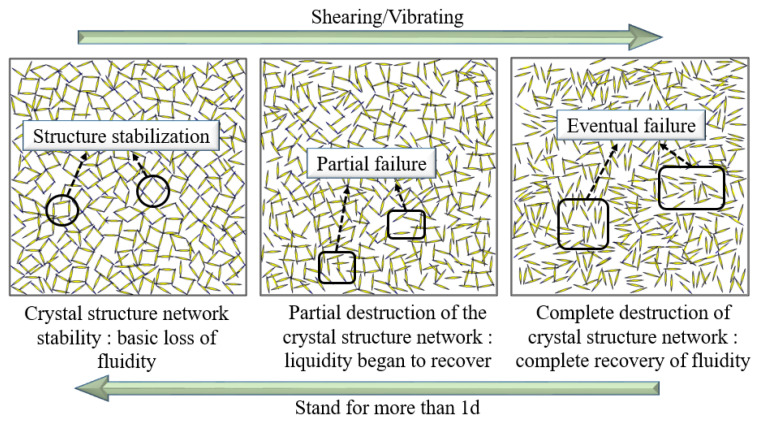
Decomposition and remodeling of the thixotropic structure of the B liquid.

The solubility of CaSO_4_·2H_2_O in water is lower, so as the hydration reaction of CaSO_4_·0.5H_2_O proceeds, CaSO_4_·2H_2_O colloidal particles are continuously precipitated from the solution. The free water in the slurry gradually decreases, and the viscosity of the slurry increases [35,36,37]. After the thickening period progresses into the false condensation period, the colloidal particles began to coalesce into crystals and the crystals gradually grow, symbiotic and interlaced, leading to a significant loss of fluidity in the system. At this stage, CaSO_4_·0.5H_2_O solubility gradually decreases with the rise in temperature, and the difference in solubility between CaSO_4_·0.5H_2_O and CaSO_4_·2H_2_O consequently decreases, leading to a gradual slowdown in the crystallization of CaSO_4_·2H_2_O [38]. As the hydration reaction progresses, the viscosity continues to increase until it reaches its peak. At this point, the crystallization rate of CaSO_4_·2H_2_O significantly decreases, unable to repair the crystal structure network destroyed by shear due to rotor rotation. Macroscopically, it manifests as a decrease in slurry viscosity and a gradual restoration of fluidity. As shown in Figure 5, the B liquid was thixotropic and reversible, and when the rotor rotated, the CaSO_4_·2H_2_O crystal particles were gradually dispersed, the viscosity of the slurry decreased, and the fluidity was gradually restored. When left to stand for a long time, the crystal particles gradually flocculated due to colloidal gravity, the interacting particles formed an osmotic network, the viscosity of the slurry rose, and the fluidity was gradually lost. After the end of the false condensation period into the stabilization period, this period of time slurry mobility was completely restored and the downward trend in viscosity gradually smoothed, finally reaching a stable value. At this time, the high content of CaSO_4_·2H_2_O in liquid B was able to quickly react with C_3_A to form AFt when mixed with liquid A, thus accelerating the hydration rate of SACDL, as shown by Formula (2). By prolonging the B-liquid mixing time in the grouting project and carrying out two-liquid mixing during the B liquid’s stabilization period, we found that the pumpability of the slurry can be improved to avoid problems such as grouting pipe plugging.

(2) $$C_{3}A+3CSH_{2}+26H\to C_{6}AS_{3}H_{32}$$

**Figure 6:**
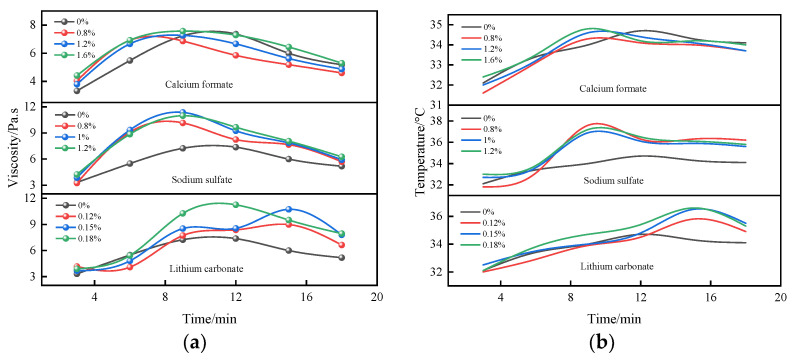
Time-varying characteristic curve of the viscosity and temperature of liquid B under the action of a single-mixed early strength agent. (**a**) The viscosity of liquid B under the action of a single early strength agent; (**b**) the temperature of liquid B under the action of a single early strength agent.

Figure 6 shows the time-varying characteristic curves of the viscosity and temperature of the B liquid under the action of single doping of the early strength agent. The doping of the early strength agent has a significant effect on the viscosity and temperature of the B liquid. As can be seen from Figure 6, both calcium formate and sodium sulfate are able to shorten the duration of the thickening period of the B liquid so that the slurry enters the pseudo-setting period earlier. The viscosity and temperature of the slurry increased and then decreased with the increase in sodium sulfate doping, and the viscosity and temperature of the slurry peak increased significantly, which was due to the hydration of quicklime and slaked lime in the ethanol to increase the concentration of CH in the liquid phase. This prompted a reaction between sodium sulfate and CH to generate CaSO_4_ with very high activity, thus accelerating the generation rate of CaSO_4_·2H_2_O colloidal particles and causing the viscosity and temperature of the slurry to increase rapidly [39,40]. When the dosage of sodium sulfate was 1%, the viscosity of the slurry reached up to 11.3 Pa.s, at which time the temperature rose to 37 °C. Contrary to sodium sulfate, single doping of lithium carbonate can significantly extend the duration of thickening period of liquid B. This is because lithium carbonate easily reacts with CaSO_4_·2H_2_O to generate CaCO_3_, resulting in a decrease in the concentration of calcium ions in the liquid phase [41] as well as the early inability to generate a stable CaSO_4_·2H_2_O colloid. After the reaction of lithium carbonate was complete, liquid B gradually entered the pseudo-coagulation period, during which it underwent an exothermic reaction.

**Figure 7:**
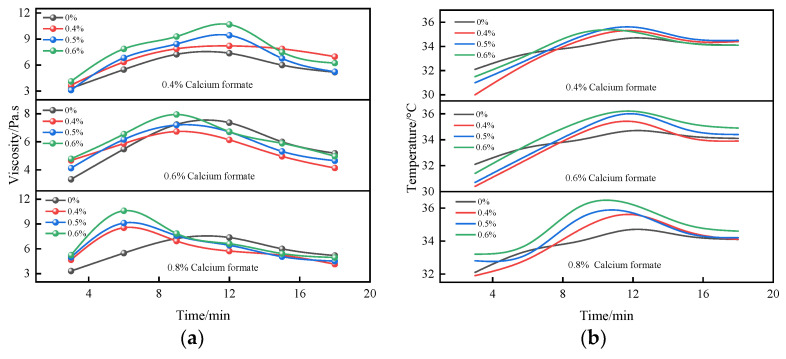
Time-varying characteristic curve of viscosity and temperature of liquid B under the action of composite early strength agent; (**a**) B liquid’s viscosity under the effect of a composite early strength agent; (**b**) B liquid’s temperature under the action of a compound early strength agent.

As seen in Figure 7, under the combined action of calcium formate and sodium sulfate, the thickening time of the solution gradually shortened with the increase in the calcium formate content. The compounding of 0.4% calcium formate with sodium sulfate significantly increased the viscosity of the B liquid, and the viscosity and temperature of the slurry increased with the increase in sodium sulfate dosing. When the dosage of calcium formate was increased to 0.6%, the addition of sodium sulfate caused the viscosity of liquid B to decrease, and at the same time shortened the thickening period of the B liquid. When the dosage of calcium formate reached 0.8%, the slurry thickening period was shortened to 4 min, and the false condensation period was also significantly shortened compared with the blank group. The viscosity and temperature of the slurry reached their peak values in advance compared with that of a low dosage of the composite early strength agent. This is because the hydration reaction rate of the B liquid is mainly controlled by the concentration of SO_4_^2−^ and Ca^2+^ in the liquid phase, and under the action of a high dosage of the composite early strength agent, the concentration of SO_4_^2−^ and Ca^2+^ in the liquid phase greatly increased. This resulted in an acceleration of the generation of CaSO_4_·2H_2_O colloids and the growth rate of the crystal [42], greatly shortening the period of B liquid thickening and the false condensation period. This tended to stabilize the value. In addition, the conversion of CaSO_4_·0.5H_2_O into CaSO_4_·2H_2_O was accompanied by an exothermic effect, resulting in a rapid increase in temperature and allowing liquid B to enter the pseudo-condensation period.

### 3.2. XRD Analysis of Hydration Products

**Figure 8:**
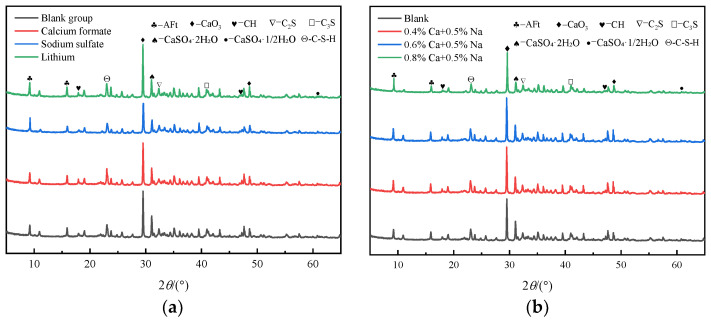
XRD spectrum of SACDL, hydrated for 3 h under the action of an early strength agent. (**a**) Single-doped early strength agent; (**b**) composite early strength agent.

Figure 8 shows the XRD pattern of SACDL, hydrated for 3 h under the effect of the early strength agents. As can be seen from Figure 8a, compared with the blank group, the incorporation of calcium formate resulted in an increase in the intensity of the diffraction peaks of the hydrated minerals, such as C-S-H and CH, in the material, and a slight decrease in the intensity of the diffraction peaks of C_3_S, C_2_S, and CaSO_4_·2H_2_O, which was attributed to the adsorption-permeation effect of calcium formate accelerating the early hydration rate of SACDL. On the one hand, calcium formate increased the Ca^2+^ concentration in the liquid phase, and the adsorption of Ca^2+^ on C_3_S promoted the hydration of C_3_S and C_2_S and accelerated the rate of C-S-H and CH generation [43]. On the other hand, the diffusion rate of HCOO^−^ was much higher than that of Ca^2+^, and was able to penetrate into the hydration layer of C_3_S and C_2_S, facilitating the dissolution of C_3_S and C_2_S [44]. The relative peak intensities of SACDL-hydrated minerals under the effect of sodium sulfate were weakened compared with those of the blank group, in which the CH peak was weak, the intensities of CaCO_3_ and CaSO_4_·2H_2_O diffraction peaks were significantly reduced, and the intensity of the AFt diffraction peak was increased. This is because sodium sulfate readily undergoes a complex decomposition reaction with CH in the liquid phase to form CaSO_4_ and NaOH, as shown in Equation (3):

(3) $$Na_{2}SO_{4}+Ca{\left(OH\right)}_{2}\to CaSO_{4}↓+2NaOH$$

This CaSO_4_ is fine, highly reactive, and can react rapidly with C_3_A to produce large amounts of AFt, which in turn leads to a decrease in CaCO_3_ production [22]. And NaOH, as an active agent, can increase the solubility of C_3_A and CaSO_4_·2H_2_O, which may be one of the reasons for the decrease in the relative diffraction peak intensity of CaSO_4_·2H_2_O [45]. Unlike sodium sulfate, under the precipitation polarization effect of lithium carbonate, the intensities of the CaCO_3_ and AFt diffraction peaks were significantly increased compared with those of the blank group, because lithium carbonate was able to consume the free Ca^2+^ in the liquid phase to generate CaCO_3_ precipitation. This lowered the concentration of CH in the liquid phase and increased the solid-phase ratio in the slurry while promoting the generation of AFt. In addition, Li^+^ has a small radius and strong polarization, which can accelerate the rupture of the hydration protective film on the surface of cement particles and increase the contact area of C_2_S and C_3_S with water, thus accelerating the hydration process [25].

The 3 h XRD patterns of SACDL, hydrated under the compounding of different dosages of calcium formate and 0.5% sodium sulfate, are shown in Figure 8b, and the intensity of the AFt diffraction peaks slightly increased when the dosage of calcium formate was 0.4% (0.4%Ca + 0.5%Na) or 0.6% (0.6%Ca + 0.5%Na), and the intensity of its diffraction peaks increased gradually with the increase in calcium formate dosage. When the dosage of calcium formate was increased to 0.8% (0.8%Ca + 0.5%Na), the intensity of the AFt diffraction peak reached its highest point, while the intensity of the CaSO_4_·2H_2_O diffraction peak decreased. This was attributed to the fact that the lower dosage of the composite early strength agent increased the solubility of C_3_A and CaSO_4_·2H_2_O, and the presence of a large amount of CaSO_4_·2H_2_O in the slurry promoted the hydration of C_3_A to generate AFt precipitation, as shown in Equation (2). With the increase in calcium formate doping, SACDL hydration was further accelerated, which was attributed to the further increase in Ca^2+^ concentration in the liquid phase. This, on the one hand, increased the saturation indices of C-S-H gel and AFt and improved its precipitation chemical driving force, which in turn promoted the hydration degree of SACDL; on the other hand, it easily reacted with SO_4_^2−^ dissociated from sodium sulfate to form more reactive CaSO_4_, thus speeding up the reaction with C_3_A to form AFt.

### 3.3. Observation of Microscopic Morphology of Hydration Products

**Figure 9:**
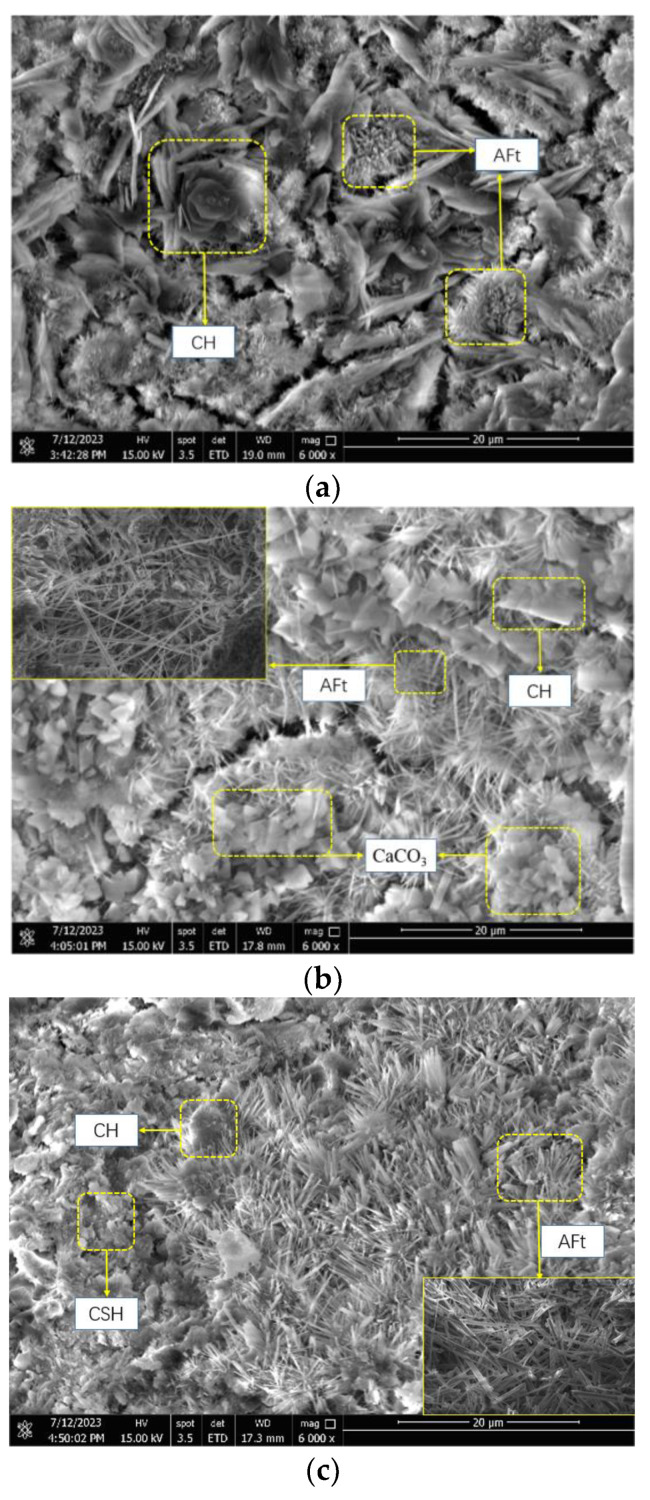
Microstructure of SACDL hydrated for 3 h under the action of an composite early strength agent. (**a**) Blank group; (**b**) 0.4% calcium formate + 0.5% sodium sulfate; (**c**) 0.8% calcium formate + 0.5% sodium sulfate.

The microscopic morphology of SACDL when hydrated for 3 h under the effect of the composite early strength agent is shown in Figure 9. As seen in Figure 9a, more microcracks in the blank group and a large amount of CH in the form of hexagonal plates can be seen from them; this was due to the dissolution of CaSO_4_·0.5H_2_O causing SO_4_^2−^ in the liquid phase to be saturated. This inhibited the growth of the CH{001} facies group, resulting in mostly plate-like CH [46]. Its layered structure and sheet shape made it difficult for the structure to provide a hard skeleton role, leading to a negative impact on the strength of SACDL. When the content of calcium formate was 0.4% and the content of sodium sulfate was 0.5%, a large amount of CaCO_3_ and elongated needle-like AFt could be observed, and microcracks were reduced. This was due to the early reaction of sodium sulfate with CH in the liquid phase. CaSO_4_ with higher activity was provided and allowed to react with C_3_A to form AFt. Calcium formate reduced the system pH and improved the rate of C_3_S hydration, in addition to increasing the concentration of Ca^2+^ in the liquid phase. This promoted the generation of AFt [23], which corresponds to the results of the XRD tests. When the dosage of calcium formate was increased to 0.8%, SACDL was hydrated to a higher degree and had a denser structure, which was attributed to the fact that the further increase in Ca^2+^ concentration promoted the nucleation and precipitation of CH. This, in turn, accelerated the formation of C-S-H and facilitated the increase in the generation of AFt, and the shape was transformed from elongated needles to rods. At this time, the SO_4_^2−^ concentration in the hydration reaction decreased; the generation of hexagonal plate-like CH decreased; and a large number of rod-like AFt crystals were interlaced with each other with C-S-H filling in between, which dramatically improved the early strength of SACDL.

## 4. Conclusions

(1) The adsorption–permeation effect of calcium formate, the complex decomposition reaction of sodium sulfate, the precipitation polarization effect of lithium carbonate, and the synergistic effects of composite early strength agents can accelerate the early hydration rate of SACDL, shorten the setting time of SACDL slurry while ensuring the fluidity of the B liquid, and improve its early strength. Our comprehensive analysis shows that the composite early-strength agent had the best effect on the modification of SACDL; 0.8% calcium formate was compounded with 0.5% sodium sulfate to promote coagulation. The early-strength effect was significant; the initial and final coagulation durations of SACDL were shortened to 5 min and 10 min, respectively; and the compressive strength of 3 h reached up to 16.7 MPa, 31% higher than that of the blank group.
(2) The composite early strength agent was able to simultaneously increase the concentrations of SO_4_^2−^ and Ca^2+^ in the liquid phase and accelerate the formation of the CaSO_4_·2H_2_O colloid and the crystal growth rate, thereby shortening the thickening period and pseudo-condensation period of the B liquid. Under the combined action of 0.8% calcium formate and 0.4% sodium sulfate, the viscosity of liquid B tended to be stable within 9 min, effectively shortening the mixing times of liquid A and liquid B and greatly improving the efficiency of double liquid grouting engineering.
(3) The solubility of C_3_A and CaSO_4_·2H_2_O increased under the condition of a low-doped composite early strength agent, which increased the rate of AFt generation. HCOO^−^ was able to penetrate into the hydration layer of C_3_S and C_2_S and to accelerate the dissolution of C_3_S and C_2_S, which, thus, jointly promoted the early hydration of SACDL.
(4) Under the condition of a high dosage of the composite early strength agent, AFt was transformed from an elongated needle-like to a rod-like shape. A further increase in the Ca^2+^ concentration promoted the crystallization nodules and precipitation of CH. The C-S-H gel was rapidly formed and filled the gaps between a large number of rod-like AFt crystals, resulting in a denser structure and effectively improving the early strength of SACDL.

## Figures and Tables

**Table 1:** Chemical composition.

Composition	Al_2_O_3_	SiO_2_	Fe_2_O_3_	CaO	MgO	TiO_2_	SO_3_	H_2_O	Other
SAC/wt%	29.72	9.90	3.99	42.36	1.32	1.33	9.77	—	1.61
Gypsum/wt%	0.41	2.14	0.18	34.85	0.21	—	48.83	10.75	2.63

**Table 2:** Liquid A and liquid B ratios.

Liquid A	SAC	H_2_O	VAE	HPMC	PBS	BX	SCE
	1	0.45	0.2%	0.1%	0.15%	0.3%	0.4%
Liquid B	CaSO_4_·0.5H_2_O	Quicklime	Slaked lime	H_2_O	VAE	HPMC	PBS
	0.8	0.1	0.1	0.45	0.2%	0.1%	0.2%

**Table 3:** Composite group test factors and levels.

Level/Factor	Sodium Calcium Formate Dosage/%	Sodium Sulfate Dosage/%
A_1_	0.4	0.4
A_2_	0.6	0.5
A_3_	0.8	0.6
